# Draft Genome Sequences of 11 Lactobacillus jensenii Strains Isolated from the Female Bladder

**DOI:** 10.1128/MRA.00970-19

**Published:** 2019-08-29

**Authors:** Catherine Putonti, Ayesha Ahmad, Genevieve Baddoo, Jessica Diaz, Michele Do, Noreen Gallian, Collin Lorentzen, Heena Mohammed, Jodi Murphy, Adetokunbo Olu-Ajeigbe, Taylor Yang, Taylor Miller-Ensminger, Nicole Stark, Laura Maskeri, John Van Dusen, Alan J. Wolfe

**Affiliations:** a Bioinformatics Program, Loyola University Chicago, Chicago, Illinois, USA; b Department of Biology, Loyola University Chicago, Chicago, Illinois, USA; c Department of Computer Science, Loyola University Chicago, Chicago, Illinois, USA; d Department of Microbiology and Immunology, Stritch School of Medicine, Loyola University Chicago, Maywood, Illinois, USA; e Department of Chemistry and Biochemistry, Loyola University Chicago, Chicago, Illinois, USA; Georgia Institute of Technology

## Abstract

Lactobacillus jensenii, a protective bacterium in the vaginal microbiota, is also a member of the female urinary tract community. Here, we report 11 genome sequences of L. jensenii strains isolated from catheterized urine from women. This effort greatly increases our knowledge of the genetic diversity of this species within the bladder.

## ANNOUNCEMENT

Lactobacillus jensenii is one of the many Lactobacillus species that dominates the healthy female genital tract (1) and one of three *Lactobacillus* species found in both the vaginal and bladder microbiota (2). Prior studies have found that L. jensenii has a bactericidal effect that prevents viral infection and the colonization of bacteria in the female vagina and bladder, including that by the uropathogen Escherichia coli (3). Despite the important role of L. jensenii in these two microbiota, only two strains to date have been characterized from the bladder (2). In an effort to more fully characterize this commensal bacterium, we have isolated numerous strains of L. jensenii from catheterized urine and voided urine samples from women (4–8) and recently sequenced a subset of this collection.

Catheterized urine samples collected from women as part of prior institutional review board (IRB)-approved studies (4–8) were cultured using the enhanced quantitative urine culture (EQUC) method (8) and stored at –80°C. From these samples, 11 L. jensenii strains, identified by matrix-assisted laser desorption ionization–time of flight (MALDI-TOF) mass spectrometry, were selected for whole-genome sequencing. Each strain was first streaked on a Columbia colistin-nalidixic acid (CNA) agar with 5% sheep blood plate (catalog number 221353; BD) and incubated at 35°C in 5% CO_2_ for 48 hours. A single colony was selected and grown in MRS liquid medium at 35°C in 5% CO_2_ for 48 hours. DNA was extracted using the Qiagen DNeasy UltraClean microbial kit and quantified using a Qubit fluorometer. DNA libraries were created using the Nextera XT library prep kit and sequenced using the MiSeq reagent kit v2, producing, on average, 742,756 pairs of 250-bp reads. Raw reads were trimmed using Sickle v1.33 (https://github.com/najoshi/sickle) and assembled using SPAdes v3.13.0 (9) with the “only-assembler” option for k = 55, 77, 99, and 127. CheckM v1.0.12 (10) was used to evaluate the genome assembly completeness and contamination. Genome coverage was calculated using BBMap v38.47 (https://sourceforge.net/projects/bbmap/). The NCBI Prokaryotic Genome Annotation Pipeline (PGAP) v4.8 (11) was used to annotate the genome sequences. CRISPR arrays were identified using CRISPRCasFinder v1.1.1 (12). Unless previously noted, default parameters were used for each software tool.

Table 1 lists the genome assembly statistics for the 11 bladder L. jensenii strains. The average GC content is 34.4%, similar to that of other strains for the species in GenBank. Annotations identified an average of 1,554 coding sequences (CDS) (Table 1). The strains vary in their number of rRNA operons and tRNAs. Strains UMB7846 and UMB8354 presented the greatest challenges during assembly, with *N*_50_ values that were significantly smaller than those of the other strains. This difficulty has been previously noted for the genus and is due to its numerous short repeats (13). Nonetheless, genome completeness, as predicted by CheckM, was 98.4% and 99.9%, respectively. The genome assembly for L. jensenii UMB1303 was found to encode only 17 tRNAs. This is significantly fewer than those encoded by the other bladder L. jensenii genomes and genomes from vaginal L. jensenii strains and thus warrants further investigation. CRISPR arrays were identified in five of the strains, UMB0034, UMB1165, UMB7846, UMB8354, and UMB8489. Only two other L. jensenii strains, SNUV360 (GenBank accession number CP018809) and JV-V16 (GenBank accession number CM000953), have CRISPR arrays recorded in the CRISPR-Cas database (12).

**TABLE 1:** Genome assembly statistics

Strain	Coverage (×)	No. of contigs	*N*_50_ (bp)	Genome length (bp)	No. of CDS^*a*^	No. of rRNAs	No. of tRNAs	GenBank WGS^*b*^ accession no.	SRA^*c*^ accession no.
UMB0034	185.33	58	51,483	1,608,111	1,556	3	56	VNGB00000000	SRR9695706
UMB0037	483.25	65	58,772	1,677,685	1,659	3	56	VNGA00000000	SRR9695716
UMB0055	152.42	73	51,485	1,616,563	1,589	3	56	VNFZ00000000	SRR9695711
UMB0732	47.77	254	8,084	1,444,087	1,503	3	53	VNFR00000000	SRR9695705
UMB1165	130.83	54	53,869	1,690,640	1,667	3	59	VNGH00000000	SRR9695717
UMB1303	59.46	81	21,419	1,255,994	1,183	2	17	VNGF00000000	SRR9695709
UMB1307	60.25	83	31,156	1,610,679	1,509	2	38	VNGG00000000	SRR9695718
UMB1355	189.76	57	78,618	1,730,238	1,637	3	53	VNGE00000000	SRR9695708
UMB7846	48.33	629	3,050	1,517,837	1,741	3	45	VNFP00000000	SRR9695710
UMB8354	77.77	528	3,321	1,335,500	1,462	1	47	VNFO00000000	SRR9695715
UMB8489	68.55	94	31,778	1,657,936	1,592	3	56	VNFW00000000	SRR9695722

a CDS, coding sequences.

b WGS, whole-genome shotgun.

c SRA, Sequence Read Archive.

Prior to this study, only two L. jensenii strains from the bladder had been sequenced (2). Thus, the addition here of 11 genomes greatly increases our knowledge of the genetic diversity of this beneficial member of the bladder microbiota.

### Data availability.

This whole-genome shotgun (WGS) project has been deposited in GenBank, and the accession numbers for each genome assembly are listed in Table 1. The versions described in this paper are the first versions. Raw sequence data are publicly available for the 11 L. jensenii strains in the SRA; the accession numbers are listed in Table 1.
